# SMILE Downregulation during Melanogenesis Induces MITF Transcription in B16F10 Cells

**DOI:** 10.3390/ijms232315094

**Published:** 2022-12-01

**Authors:** Xuan T. Truong, Young-Seung Lee, Thuy T. P. Nguyen, Hyun-Jin Kim, Sung-Hak Kim, Changjong Moon, Don-Kyu Kim, Hueng-Sik Choi, Tae-Il Jeon

**Affiliations:** 1 Department of Animal Science, Chonnam National University, Gwangju 61186, Republic of Korea; 2 Department of Veterinary Anatomy and Animal Behavior, College of Veterinary Medicine and BK21 FOUR Program, Chonnam National University, Gwangju 61186, Republic of Korea; 3 Department of Integrative Food, Bioscience, and Biotechnology, Chonnam National University, Gwangju 61186, Republic of Korea; 4 School of Biological Sciences and Technology, Chonnam National University, Gwangju 61186, Republic of Korea

**Keywords:** cAMP, melanogenesis, MITF, skin pigmentation, SMILE

## Abstract

SMILE (small heterodimer partner-interacting leucine zipper protein) is a transcriptional corepressor that potently regulates various cellular processes such as metabolism and growth in numerous tissues. However, its regulatory role in skin tissue remains uncharacterized. Here, we demonstrated that SMILE expression markedly decreased in human melanoma biopsy specimens and was inversely correlated with that of microphthalmia-associated transcription factor (MITF). During melanogenesis, α-melanocyte-stimulating hormone (α-MSH) induction of MITF was mediated by a decrease in SMILE expression in B16F10 mouse melanoma cells. Mechanistically, SMILE was regulated by α-MSH/cAMP/protein kinase A signaling and suppressed MITF promoter activity via corepressing transcriptional activity of the cAMP response element-binding protein. Moreover, SMILE overexpression significantly reduced α-MSH-induced MITF and melanogenic genes, thereby inhibiting melanin production in melanocytes. Conversely, SMILE inhibition increased the transcription of melanogenic genes and melanin contents. These results indicate that SMILE is a downstream effector of cAMP-mediated signaling and is a critical factor in the regulation of melanogenic transcription; in addition, they suggest a potential role of SMILE as a corepressor in skin pigmentation.

## 1. Introduction

Melanin is a pigment that colors the skin, hair, and eyes, and protects the skin from damage and aging caused by UV irradiation [1]. However, excessive melanin production or deposition can lead to a wide range of hyperpigmentation disorders, such as melasma, actinic and senile lentigines, and post-inflammatory hyperpigmentation, or even skin cancers [2]. Hyperpigmentation is generally benign; however, it often requires frequent evaluations by a dermatologist and affects the quality of life of patients. Melanin generation (i.e., melanogenesis) in melanocytes is regulated by tyrosine via the activity of tyrosinase (TYR) and tyrosinase-related proteins (TRPs) [3]. During melanogenesis, α-melanocyte-stimulating hormone (α-MSH), a keratinocyte peptide secreted in response to UV irradiation, activates the cyclic adenosine monophosphate (cAMP)-protein kinase A (PKA) pathway through melanocortin receptor 1 (MC1R), which in turn induces microphthalmia-associated transcription factor (MITF), a key transcriptional regulator of enzymes involved in melanogenesis, such as TYR and TRPs [3,4]. MITF can also regulate the proliferation and differentiation of melanocytes and MITF mutations, which are associated with phenotypes of Waardenburg syndrome, such as deafness and hypopigmentation in humans [5]. Therefore, characterizing the intracellular mechanisms underlying the regulation of MITF expression and activity would provide key insights into the dynamics of skin pigmentation.

The transcription of MITF is controlled by a number of transcription factors and multiple signaling events involved in melanogenesis, among which the cAMP/PKA pathway is widely known to be the most critical [6]. The cAMP/PKA pathway induces cAMP-responsive element-binding protein (CREB) phosphorylation, which robustly activates MITF transcription [3]. The sex-determining region Y-box 10 (SOX10) cooperates with CREB for optimal MITF expression [7]. Furthermore, it was reported that peroxisome proliferation-activated receptor gamma coactivator-1 (PGC-1) coactivators induce the activity of the MITF promoter via coactivation of SOX10 in α-MSH-induced melanogenesis. α-MSH induces PGC-1α mRNA expression while reducing the degradation of both PGC-1α and PGC-1β proteins; thus, prolonging the transcriptional activity of SOX10 on the MITF promoter to stimulate melanogenesis [8]. However, the regulation of the MITF promoter is complex and not completely understood.

CREB and activating transcription factor (ATF) basic leucine zipper (bZIP) transcription factor (CREBZF), also known as the small heterodimer partner (SHP)-interacting leucine zipper protein (SMILE), belongs to the CREB/ATF gene family. However, unlike other family members, it lacks DNA-binding activity as a homodimer [9,10]. Recently, SMILE was reported to function as a critical corepressor in liver glucose metabolism. For example, insulin-induced SMILE competed for PGC-1α to inhibit hepatocyte nuclear factor 4α promoter activity and suppress the gluconeogenic program in the liver [11]. Additionally, SMILE suppresses the progression of several types of cancer, such as breast, gastric, and prostate cancer via the regulation of a broad transcriptional program [12,13,14]. However, the role of SMILE in skin pigmentation, particularly in melanogenesis, is completely unknown. In this study, we demonstrated that SMILE negatively regulates melanogenesis via the inhibition of MITF transcription.

## 2. Results

### 2.1. SMILE Inversely Correlates with MITF in Human Melanoma Biopsies

**Figure 1:**
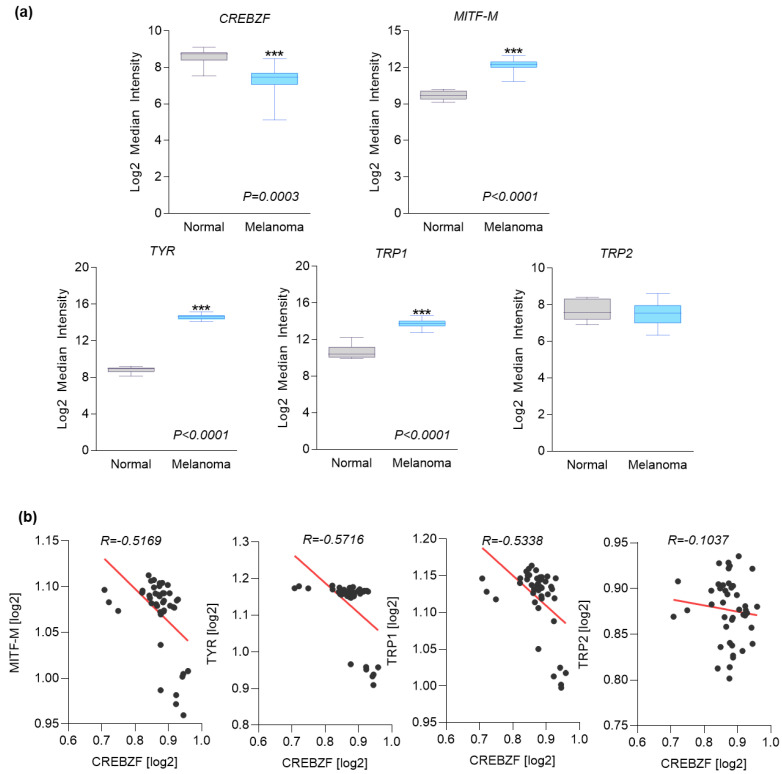
SMILE inversely correlates with MITF in human melanoma biopsies. (**a**) Expression level of CREBZF, MITF, TYR, TRP1, and TRP2 in human normal (*n* = 7) and melanoma (*n* = 34) biopsies (GSE3189). The boxes represent the 25th to 75th percentiles, the line is at the median, and the whiskers represent the range. *** *p* < 0.0005 vs. Normal. (**b**) Negative correlation between CREBZF and melanogenic genes in human melanoma (*n* = 34) biopsies (GSE3189). *p* = 0.0004 vs. MITF-M; *p <* 0.0001 vs. TYR; *p* = 0.0002 vs. TRP1; *p* = 0.5080 vs. TRP2.

To investigate the role of SMILE in melanogenesis, we first compared the expression level of SMILE to those of melanogenic genes in human melanoma samples characterized by the presence of melanin using an available dataset (GSE3189). Interestingly, SMILE was significantly downregulated in melanoma tissues (Figure 1a, *p* = 0.0003) compared to normal tissues, whereas the expression of the melanocyte-specific form of MITF (MITF-M) and its melanogenic target genes such as TYR and TRP1 was upregulated as expected (Figure 1a). However, the expression of TRP2 was similar between the normal and melanoma condition (Figure 1a), which may be caused by the indirect transactivation of MITF on the TRP2 gene promoter [15]. Moreover, we found that SMILE was inversely correlated with the expression level of the melanogenic genes except TRP2 in melanoma tissues on the same dataset (Figure 1b), suggesting that SMILE may be a negative regulator of melanin production.

### 2.2. SMILE and MITF Are Reciprocally Regulated by α-MSH in B16F10 Mouse Melanoma Cells

**Figure 2:**
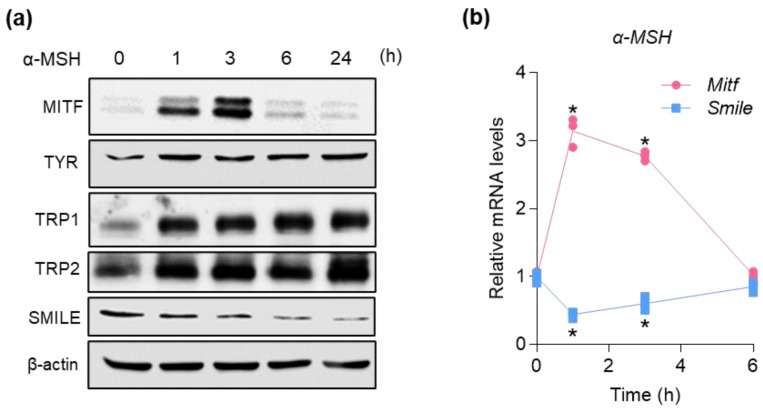
SMILE and MITF are reciprocally regulated by α-MSH in B16F10 mouse melanoma cells. Cells were treated with 1 µM α-MSH in a time-dependent manner. (**a**) Protein levels of MITF, melanogenic enzymes, and SMILE. (**b**) mRNA levels of genes encoding SMILE and MITF. * *p* < 0.05 vs. no treatment of α-MSH by one-way ANOVA.

To test whether SMILE expression is regulated during melanogenesis, B16F10 melanotic murine melanoma cells were treated with α-MSH, after which we measured the expression of SMILE and melanogenic genes during melanogenesis. MITF protein levels rapidly increased during the first hour of α-MSH treatment; they then decreased after 6 h, followed by an upregulation of the melanogenic enzymes TYR, TRP1, and TRP2 in a time-dependent manner until 24 h in B16F10 cells (Figure 2a). Similarly, the mRNA expression levels of MITF also tended to rise and then, decrease to the basal level in cells (Figure 2b). In contrast, the expression levels of both mRNA and protein of SMILE were rapidly and markedly decreased after 1 h; then, the SMILE mRNA levels were recovered to basal levels at 6 h after α-MSH treatment in cells (Figure 2), indicating a reciprocal regulation of SMILE and MITF during melanogenesis.

### 2.3. SMILE Downregulates Melanogenesis in Melanoma Cells

**Figure 3:**
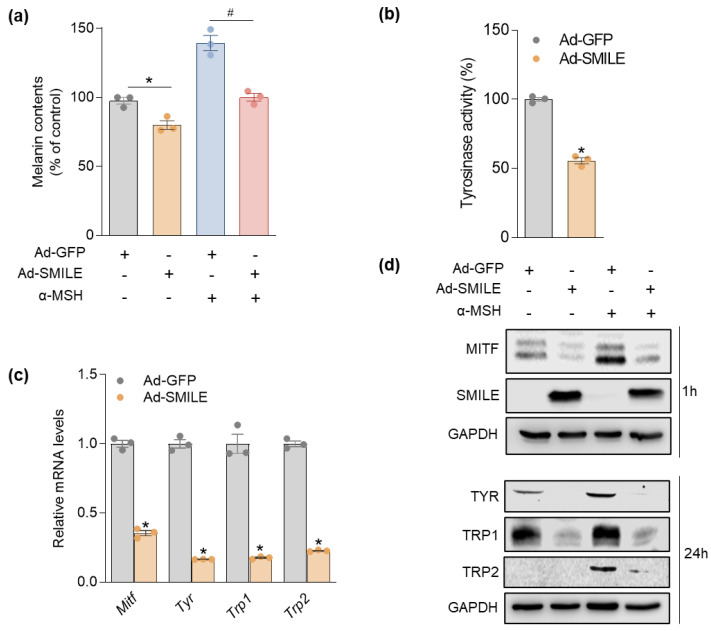
SMILE overexpression downregulates the expression of melanogenic genes in B16F10 cells. Cells were infected with Ad-GFP or Ad-SMILE for 48 h and then, treated with 1 µM α-MSH for 1 h or 24 h. (**a**) Melanin contents. (**b**) Cellular tyrosinase activity. (**c**) mRNA levels of melanogenic genes. (**d**) Protein levels of SMILE and melanogenic genes. Data are mean ± SEM. * *p* < 0.05 vs. Ad-GFP; ^#^ *p* < 0.05 vs. Ad-GFP + α-MSH by one-way ANOVA (**a**). * *p* < 0.05 vs. Ad-GFP (**b**,**c**) by Student’s *t*-test.

Our results demonstrated an inverse correlation between SMILE and melanogenic genes. Therefore, we next examined whether SMILE, a transcriptional corepressor, could regulate melanin synthesis and melanogenic gene expression. B16F10 cells were infected with adenovirus expressing SMILE or control GFP. As shown in Figure 3a,b, the overexpression of SMILE markedly suppressed cellular melanin contents and tyrosinase activity under both basal and α-MSH-stimulated culture conditions compared to those in control adenovirus-infected cells. Moreover, the ectopic expression of SMILE in B16F10 cells resulted in more than 50% reduction of mRNA and protein levels of melanogenic genes, including MITF, TYR, TRP1, and TRP2 (Figure 3c,d).

**Figure 4:**
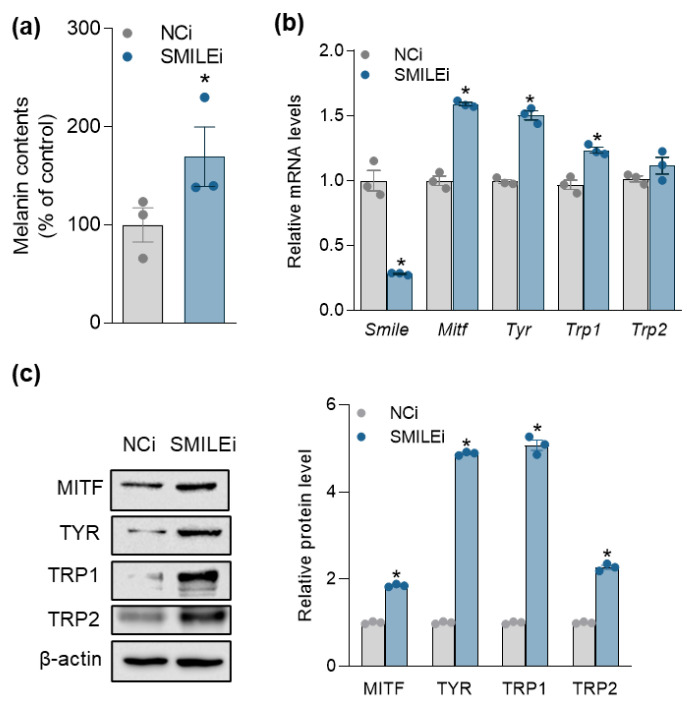
SMILE knockdown upregulates the expression of melanogenic genes in B16F10 cells. Cells were transfected with siRNA negative control (NCi, 50 nM) or siRNA targeting SMILE (50 nM) for 48 h. (**a**) Melanin contents. (**b**) mRNA and (**c**) protein levels of SMILE and melanogenic genes. Data are mean ± SEM. * *p* < 0.05 vs. NCi (**a**,**b**) by Student’s *t*-test.

Conversely, the silencing of SMILE by small interfering RNA (siRNA) significantly increased cellular melanin contents (Figure 4a), and mRNA and protein levels of melanogenic genes in B16F10 cells (Figure 4b,c). These findings suggest that SMILE downregulation is required for the induction of melanogenic genes during α-MSH-stimulated melanogenesis in mouse melanotic melanoma cells.

### 2.4. SMILE Suppresses Transcription of MITF via Binding to CREB

**Figure 5:**
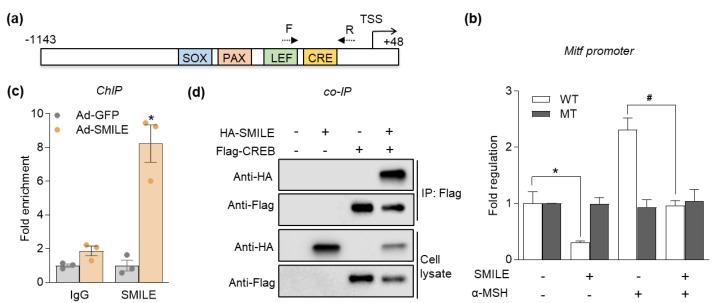
SMILE suppresses transcription of MITF via binding to CREB. (**a**) Cis-regulatory elements bound by indicated transcription factors on moue Mitf promoter. (**b**) B16F10 cells were co-transfected with pGL3-Mitf-M (wtCRE or mtCRE) and/or pcNDA3.1 Flag-SMILE for 48 h, after which the cells were treated with 1 µM α-MSH for 1 h. Luciferase reporter assays were performed for measuring promoter activity. (**c**) ChIP assay for SMILE binding to the CRE site of the Mitf promoter. IgG was used as the negative control. (**d**) HA-SMILE and Flag-CREB were co-transfected into 293T cells for 48 h. IP was performed with Flag antibodies and analyzed with HA antibodies. Data are mean ± SEM. * *p* < 0.05 vs. control; ^#^ *p* < 0.05 vs. α-MSH by one-way ANOVA (**b**). * *p* < 0.05 vs. Ad-GFP (**c**) by Student’s *t*-test.

Because MITF is a master transcription factor that controls the expression of melanogenic enzymes and is downregulated by SMILE, as shown in Figure 3, we next tested whether SMILE directly regulates MITF promoter activity. B16F10 cells were transiently transfected with a luciferase reporter vector containing Mitf promoter sequences (Figure 5a). As shown in Figure 5b, α-MSH treatment upregulated wild-type (WT) Mitf promoter activity, whereas the overexpression of SMILE significantly suppressed the effect of α-MSH.

A previous study demonstrated that SMILE suppresses the CREB transcriptional activity on the promoter of gluconeogenesis genes in nutrient-rich conditions [16], suggesting a similar regulatory role of SMILE and CREB on the MITF promoter to regulate melanogenesis. Indeed, the deletion of the CRE site in the Mitf promoter (MT) completely abolished the inhibitory effect of SMILE on basal and α-MSH-responsive promoter activities (Figure 5b), indicating that the CRE motif is important for SMILE-mediated suppression of MITF transcription. Furthermore, chromatin immunoprecipitation (ChIP) analysis revealed that SMILE binds to the region containing the CRE site on the Mitf promoter (Figure 5c) and we demonstrated that SMILE directly interacted with CREB via a co-immunoprecipitation assay (Figure 5d). Therefore, SMILE directly inhibits the activity of the MITF promoter by corepressing CREB activity.

**Figure 6:**
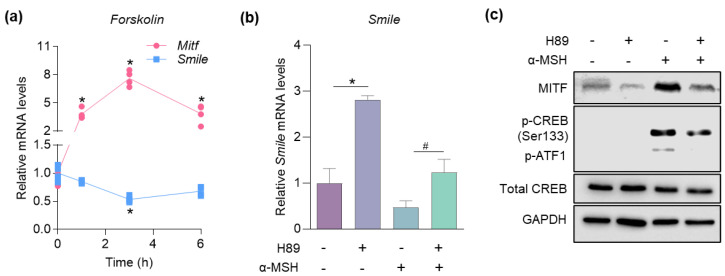
SMILE and MITF are reciprocally regulated by cAMP/PKA signaling. (**a**) B16F10 cells were treated with 10 µM forskolin in a time-dependent fashion. mRNA levels of genes encoding SMILE and MITF. (**b**,**c**) B16F10 cells were treated with 5 µM H89 for 24 h and 1 µM α-MSH for 1 h. (**b**) mRNA level of Smile and (**c**) protein levels of MITF and CREB. Data are mean ± SEM. * *p* < 0.05 vs. no treatment of α-MSH by one-way ANOVA (**a**). * *p* < 0.05 vs. control; ^#^ *p* < 0.05 vs. α-MSH by one-way ANOVA (**b**).

To elucidate the pathway through which α-MSH downregulates SMILE, B16F10 cells were treated with forskolin, cAMP activator. Consistent with the α-MSH treatment experiment (Figure 2b), the mRNA level of Mitf was markedly induced after 1 h of forskolin treatment, whereas Smile was rapidly suppressed, after which the expression of both genes returned to the basal level after 6 h of treatment (Figure 6a). Next, to test whether PKA, a cAMP-dependent kinase, is required for SMILE regulation, the cells were treated with α-MSH and the PKA inhibitor H89. Interestingly, the mRNA expression of Smile was significantly increased by H89 treatment and the inhibitory effect of α-MSH on Smile was dampened by PKA inhibition (Figure 6b). Conversely, H89 treatment decreased α-MSH-induced MITF protein levels in B16F10 cells (Figure 6c), suggesting that cAMP/PKA signaling is required for reciprocal regulation between SMILE and MITF during α-MSH-stimulated melanogenesis.

## 3. Discussion

The altered melanogenic gene expression is involved in melanogenesis dysfunction, leading to a pathological modification of skin pigmentation such as hyperpigmentation or hypopigmentation [3]. MITF is considered a master transcriptional regulator of pigmentation; in addition, melanocytic differentiation, mutations, and aberrant expression of MITF are associated with skin pigmentation abnormalities and melanoma in both mouse models and humans [17]. In this study, we identified SMILE as a negative regulator of MITF in α-MSH-induced melanogenesis in melanoma cells. SMILE acts as a coregulator that interacts with CREB to repress its transcriptional activity on the MITF promoter; thus, decreasing melanin production via the downregulation of melanogenic enzymes in mouse B16/F10 melanoma cells.

Regulation of melanogenesis is governed by multiple signaling events, among which cAMP-mediated signaling is known to be a critical pathway [18]. We found that the expression of SMILE was downregulated in response to the α-MSH or cAMP-elevating agent forskolin, which is known to promote MITF transcription. In contrast, this phenomenon is reversed by a PKA inhibitor, suggesting that the cAMP/PKA pathway drives skin pigmentation through reciprocal activation and inhibition of MITF and SMILE, respectively, during melanogenesis. Interestingly, gene expression profiling also indicated that SMILE expression is lower in human melanoma biopsy specimens than in normal tissues, and that the expression levels of SMILE and MITF were remarkably opposite, suggesting a plausible role of SMILE as a key regulator in skin pigmentation disorder or skin cancer.

A previous study reported that α-MSH induces mRNA expression of a transcriptional coactivator PGC-1α and stabilizes its protein levels through the cAMP/PKA pathway. In addition, PGC-1α directly activates the MITF promoter by coactivation of SOX10 and CREB; thus, inducing melanogenesis in melanocytes [8]. Additionally, cAMP/PKA signaling stimulates dephosphorylation of CREB-regulated transcription coactivator 3 (CRTC3) to form the CREB transcription complex, leading to MITF expression and melanogenesis [19]. Similar to PGC-1α and CRTC3, our co-IP and ChIP analysis also showed that SMILE binds to the CRE site on the MITF promoter through direct interaction with CREB, albeit suppressing the activity of the MITF promoter. Opposite expression and acting of PGC-1α and SMILE by α-MSH in melanocytes is also similar to that found in the liver during gluconeogenesis in response to glucagon signaling, which activates the CREB/CRTC2 signaling complex to induce gluconeogenic genes such as glucose-6-phosphatase and PGC-1α [11], suggesting a counteracting contribution of PGC-1α and SMILE in skin pigmentation.

In summary, our study demonstrated that SMILE negatively regulated cAMP/CREB-mediated MITF expression, which contributed to melanin production in melanocytes. Therefore, targeting SMILE in local skin could be a potential treatment for hyperpigmentation or hypopigmentation. It is also worth noting that several small molecules with antimelanogenic properties in the skin can also induce SMILE expression in other tissues [20,21,22,23]. For instance, metformin, a drug used to treat diabetes, suppresses the expression of MITF and melanogenic genes by reducing cAMP/PKA/CREB signaling, thereby inhibiting melanin production in vivo and in human reconstituted epidermis [24]. Additionally, this drug also induces SMILE expression to improve prostate cancer progression [14] and intestinal inflammation [25]. Although MITF expression is highly variable across melanoma specimens, its amplification is directly implicated in metastatic melanoma and identified as a prognostic marker for survival [6]. However, since MITF is considered as an undruggable target, targeting pathways that regulate its expression or activity could be more effective in melanoma therapy. Thus, future studies should focus on the identification of small molecules directly targeting SMILE and analysis of their therapeutic benefits.

## 4. Materials and Methods

### 4.1. Dataset Preparation for SMILE and MITF Expression Analysis

The Gene Expression Omnibus (GEO) database, including GSE3189 [26], was accessed to obtain microarray data for SMILE and MITF-related gene expression in normal and melanoma tissues. For correlation analysis, the Pearson correlation coefficient (r-value) between SMILE and each MITF-related gene was calculated using Prism 9.4.1 (GraphPad Software, San Diego, CA, USA).

### 4.2. Materials and Cell Culture

Mouse melanoma B16F10 and 293T cell lines were purchased from the American Type Culture Collection (ATCC). α-MSH (M4135), melanin (M8631), forskolin (F6886), and H89 (B1427) were obtained from Sigma-Aldrich. siRNA targeting mouse SMILE (sc-61826) and siRNA negative control (sc-37007) were purchased from Santa Cruz Biotechnology. B16F10 and 293T cells were maintained in DMEM/high glucose supplemented with 10% heat-activated fetal bovine serum (FBS) and 1% antibiotics (100 U/mL penicillin/streptomycin). The cells were cultured in a 5% CO_2_ atmosphere at 37 °C. All cell culture reagents were obtained from Gibco BRL. B16F10 cells were seeded and treated with α-MSH (1 μM), forskolin (10 μM), and H89 (5 μM) for the indicated time. The cells were also infected or transfected with Ad-hSMILE or siRNA-targeting SMILE (50 nM), respectively.

### 4.3. RNA Isolation and Analysis

Total RNA was isolated with TRIzol^TM^ (Invitrogen) according to manufacturer instructions. cDNA was synthesized using ReverTra Ace^®^ qPCR RT kit (Toyobo, Japan) and quantitative PCR was performed using an Mx3000P qPCR System (Agilent Technologies, Santa Clara, CA, USA). The primer sequences used in this study are listed in Table S1. mRNA levels were normalized to the expression of the mouse or human ribosomal protein lateral stalk subunit P0 (RPLP0), and the data were calculated via the comparative cycle threshold method.

### 4.4. Immunoblotting

The cells were harvested and lysed in RIPA buffer (Thermo Fisher Scientific, Waltham, MA, USA) containing protease and phosphatase inhibitors. The lysates were incubated for 20 min on ice, further sonicated, and centrifuged at 4 °C. The supernatants were collected and measured by using the BCA Kit (Pierce, Appleton, WI, USA). Equal amounts of protein samples were separated via SDS-PAGE and then, transferred to nitrocellulose membranes. The membranes were blocked in 5% skim milk for 1 h and incubated with primary antibodies against SMILE (NBP2-94859, Novus Biologicals, Littleton, CO, USA), MITF (MA5-14146, Thermo Fisher Scientific), p-CREB (#9198), CREB (#9197), GAPDH (#2118, Cell Signaling Technology, Danvers, MA, USA), β-actin (A5441, Sigma-Aldrich, Burlington, MA, USA), tyrosinase (sc-7833), TRP1 (sc-10443), TRP2 (sc-10451), anti-OctA probe (sc-166355), and anti-HA probe (sc-7392, Santa Cruz Biotechnology, Dallas, TX, USA) overnight at 4 °C, followed by incubation with secondary antibodies (Thermo Fisher Scientific). The blots were developed using an EZ-Western Lumi Pico or Femto Western blot detection kit (Daeil Lab Service, Seoul, Republic of Korea).

### 4.5. Luciferase Reporter Assay

The mouse MITF-M promoter constructs (pGL3-Mitf-M, wtCRE, and mtCRE) were obtained from our previous study [27]. The luciferase reporter assay was conducted in B16F10 cells. Briefly, the B16F10 cells were seeded in a 24-well plate and co-transfected for 48 h with pGL3-Mitf-M and/or pcNDA3.1 Flag-SMILE using Lipofectamine^®^2000 reagent (Invitrogen, Waltham, MA, USA), followed by α-MSH treatment for 1 h. The cells were harvested and assayed using the EZ^TM^ Luciferase Assay System (Enzynomics, Daejeon, Republic of Korea). pSV-*β-*Galactosidase control vector (Promega, Madison, WI, USA) was used as a normalization vector.

### 4.6. Determination of Melanin Content

The cells were infected with adenovirus (Ad)-SMILE or transfected with siRNA against SMILE using the Lipofectamine^TM^ RNAiMAX transfection reagent (Thermo Fisher Scientific) for 48 h; then, treated with α-MSH for 24 h. The cells were harvested by trypsinization; then, centrifugated at 1000× *g* for 5 min at 4 °C. The cell pellets were then solubilized in 1 N NaOH containing 10% DMSO at 80 °C for 1 h. The melanin content was calculated by comparing the absorbance of the samples at 405 nm with a standard curve of synthetic melanin.

### 4.7. Measurement of Tyrosinase Activity

The cells were lysed in RIPA buffer. The crude enzyme solution (20 μL; equivalent to 100 μg protein) was added to a mixture of 50 mM sodium phosphate buffer, pH 6.8 (100 μL) and 5 mM l-tyrosine (30 μL). After incubation for 3 h at 37 °C, the absorbance of the solution was measured at 475 nm.

### 4.8. Chromatin Immunoprecipitation Assay

Briefly, the B16F10 cells were washed with ice-cold PBS, cross-linked with formaldehyde (1%), and quenched with glycine (0.125 M). The samples were sonicated on ice to obtain a clear supernatant (DNA fragment size of 200–500 bp) and reverse crosslinked at 65 °C for 6 h. The samples were then pre-cleared for 2 h with protein A/G–agarose beads (Pierce) and incubated overnight with anti-SMILE. The immune complexes were pulled down using blocked protein-A/G–agarose beads. After the washing steps, DNA was purified using the QIAquick PCR purification kit (Qiagen, Hilden, Germany) and analyzed by quantitative PCR.

### 4.9. Co-Immunoprecipitation

The cells were homogenized in RIPA buffer containing protease and phosphatase inhibitors. The whole-cell lysate was centrifuged at 20,000× *g* at 4 °C for 20 min. The supernatants were collected and used for immunoprecipitation. The protein samples were pre-cleared with blocked protein A/G agarose beads with rotation at 4 °C for 2 h, followed by incubation with the indicated antibodies at 4 °C overnight. To capture the immunocomplex, the beads were added into the mixture at 4 °C for 2 h. Then, the beads were washed several times with RIPA buffer and boiled in 1 × SDS-PAGE buffer for 5 min at 95 °C.

### 4.10. Statistical Analysis

All the experiments were performed at least three times. The data were presented as the mean ± SEM. The differences between the means of the individual groups were assessed using the Student’s *t*-test or one-way analysis of variance (ANOVA, San Francisco, CA, USA); differences were considered significant at *p* < 0.05. The statistical software package Prism 9.0 (GraphPad Software, San Diego, CA, USA) was used for these analyses.

## Data Availability

Microarray datasets (GSE3189) are openly available in the Gene Expression Omnibus database at https://www.ncbi.nlm.nih.gov/geo/query/acc.cgi?acc=GSE3189 (accessed on 15 February 2021).

## Supplementary Materials

The following supporting information can be downloaded at: https://www.mdpi.com/article/10.3390/ijms232315094/s1.

## References

[1] Jablonski N.G. (2004). The evolution of human skin and skin color. Annu. Rev. Anthropol..

[2] Lee A.Y. (2021). Skin pigmentation abnormalities and their possible relationship with skin aging. Int. J. Mol. Sci..

[3] Lin J.Y., Fisher D.E. (2007). Melanocyte biology and skin pigmentation. Nature.

[4] D’Mello S.A., Finlay G.J., Baguley B.C., Askarian-Amiri M.E. (2016). Signaling pathways in melanogenesis. Int. J. Mol. Sci..

[5] Levy C., Khaled M., Fisher D.E. (2006). MITF: Master regulator of melanocyte development and melanoma oncogene. Trends Mol. Med..

[6] Hartman M.L., Czyz M. (2015). MITF in melanoma: Mechanisms behind its expression and activity. Cell. Mol. Life Sci..

[7] Huber W.E., Price E.R., Widlund H.R., Du J., Davis I.J., Wegner M., Fisher D.E. (2003). A tissue-restricted cAMP transcriptional response: SOX10 modulates alpha-melanocyte-stimulating hormone-triggered expression of microphthalmia-associated transcription factor in melanocytes. J. Biol. Chem..

[8] Shoag J., Haq R., Zhang M., Liu L., Rowe G.C., Jiang A., Koulisis N., Farrel C., Amos C.I., Wei Q. (2013). PGC-1 coactivators regulate MITF and the tanning response. Mol. Cell.

[9] Lu R., Misra V. (2000). Zhangfei: A second cellular protein interacts with herpes simplex virus accessory factor HCF in a manner similar to Luman and VP16. Nucleic Acids Res..

[10] Xie Y.B., Lee O.H., Nedumaran B., Seong H.A., Lee K.M., Ha H., Lee I.K., Yun Y., Choi H.S. (2008). SMILE, a new orphan nuclear receptor SHP-interacting protein, regulates SHP-repressed estrogen receptor transactivation. Biochem. J..

[11] Lee J.M., Seo W.Y., Han H.S., Oh K.J., Lee Y.S., Kim D.K., Choi S., Choi B.H., Harris R.A., Lee C.H. (2016). Insulin-inducible SMILE inhibits hepatic gluconeogenesis. Diabetes.

[12] Fang J., Jiang G., Mao W., Huang L., Huang C., Wang S., Xue H., Ke J., Ni Q. (2022). Up-regulation of long noncoding RNA MBNL1-AS1 suppresses breast cancer progression by modulating miR-423-5p/CREBZF axis. Bioengineered.

[13] Kim Y.J., Jeong S., Jung W.Y., Choi J.W., Hwang K.C., Kim S.W., Lee Y.C. (2020). miRNAs as potential biomarkers for the progression of gastric cancer inhibit CREBZF and regulate migration of gastric adenocarcinoma cells. Int. J. Med. Sci..

[14] Lee S.Y., Song C.H., Xie Y.B., Jung C., Choi H.S., Lee K. (2014). SMILE upregulated by metformin inhibits the function of androgen receptor in prostate cancer cells. Cancer Lett..

[15] Yasumoto K., Yokoyama K., Takahashi K., Tomita Y., Shibahara S. (1997). Functional analysis of microphthalmia-associated transcription factor in pigment cell-specific transcription of the human tyrosinase family genes. J. Biol. Chem..

[16] Lee J.M., Han H.S., Jung Y.S., Harris R.A., Koo S.H., Choi H.S. (2018). The SMILE transcriptional corepressor inhibits cAMP response element-binding protein (CREB)-mediated transactivation of gluconeogenic genes. J. Biol. Chem..

[17] Goding C.R., Arnheiter H. (2019). MITF-the first 25 years. Genes Dev..

[18] Slominski A., Tobin D.J., Shibahara S., Wortsman J. (2004). Melanin pigmentation in mammalian skin and its hormonal regulation. Physiol. Rev..

[19] Bang S., Won K.H., Moon H.R., Yoo H., Hong A., Song Y., Chang S.E. (2017). Novel regulation of melanogenesis by adiponectin via the AMPK/CRTC pathway. Pigment Cell Melanoma Res..

[20] Lee J.M., Gang G.T., Kim D.K., Kim Y.D., Koo S.H., Lee C.H., Choi H.S. (2014). Ursodeoxycholic acid inhibits liver X receptor alpha-mediated hepatic lipogenesis via induction of the nuclear corepressor SMILE. J. Biol. Chem..

[21] Lim H.Y., Kim E., Park S.H., Hwang K.H., Kim D., Jung Y.J., Kopalli S.R., Hong Y.D., Sung G.H., Cho J.Y. (2021). Antimelanogenesis effects of theasinensin A. Int. J. Mol. Sci..

[22] Kim Y.J., Kim K.S., Lim D., Yang D.J., Park J.I., Kim K.W., Jeong J.H., Choi H.S., Kim D.K. (2020). Epigallocatechin-3-gallate (EGCG)-inducible SMILE inhibits STAT3-mediated hepcidin gene expression. Antioxidants.

[23] Moon I.J., Yoo H., Paik S.H., Kim H.T., Kim S.Y., Song Y., Chang S.E. (2021). Ursodeoxycholic acid may inhibit environmental aging-associated hyperpigmentation. Antioxidants.

[24] Lehraiki A., Abbe P., Cerezo M., Rouaud F., Regazzetti C., Chignon-Sicard B., Passeron T., Bertolotto C., Ballotti R., Rocchi S. (2014). Inhibition of melanogenesis by the antidiabetic metformin. J. Investig. Dermatol..

[25] Yang S., Park J.S., Hwang S.H., Cho K.H., Na H.S., Choi J., Jhun J., Kim S.J., Lee B.I., Park S.H. (2021). Metformin-inducible small heterodimer partner interacting leucine zipper protein ameliorates intestinal inflammation. Front. Immunol..

[26] Talantov D., Mazumder A., Yu J.X., Briggs T., Jiang Y., Backus J., Atkins D., Wang Y. (2005). Novel genes associated with malignant melanoma but not benign melanocytic lesions. Clin. Cancer Res..

[27] Truong X.T., Park S.H., Lee Y.G., Jeong H.Y., Moon J.H., Jeon T.I. (2017). Protocatechuic acid from pear inhibits melanogenesis in melanoma cells. Int. J. Mol. Sci..

