# Supplementary material for: SMILE Downregulation during Melanogenesis Induces MITF Transcription in B16F10 Cells
Source: Int J Mol Sci. 2022 Dec 1;23(23):15094. doi: 10.3390/ijms232315094 (PMC9738925; doi:10.3390/ijms232315094)
Supplement: Supplementary file 1 [file ijms-23-15094-s001.zip › ijms-2041717-supplementary.pdf]

Table S1. Primer sequences used for RT-qPCR

Genes	RT-qPCR Primers	
	**Forward**	**Reverse**
Mitf-M	GCCTGAAACCTTGCTATGCTGGAA	AAGGTACTGCTTTACCTGGTGCCT
Tyr	CTCTGGGCTTAGCAGTAGGC	GCAAGCTGTGGTAGTCGTCT
Trp1	TGGGGATGTGGATTTCTCTC	AGGGAGAAAGAAGGCTCCTG
Trp2	AGGTACCATCTGTTGTGGCTGGAA	AGTTCCGACTAATCAGCGTTGGGT
Smile	GGGAGAAGAGGCGGAGAAAG	AGGTAGCGACTCTCCTCCTG
RPLP0	GTGCTGATGGGCAAGAAC	AGGTCCTCCTTGGTGAAC
ChIP primer CRE	CAGTGAGCTTGACTTTGATAGC	AATATTGATATCAGTTTCCCTGCTG
